# A retrospective study of neonatal case management and outcomes in rural Rwanda post implementation of a national neonatal care package for sick and small infants

**DOI:** 10.1186/s12887-018-1334-1

**Published:** 2018-11-12

**Authors:** Merab Nyishime, Ryan Borg, Willy Ingabire, Bethany Hedt-Gauthier, Evrard Nahimana, Neil Gupta, Anne Hansen, Michelle Labrecque, Fulgence Nkikabahizi, Christine Mutaganzwa, Francois Biziyaremye, Claudine Mukayiranga, Francine Mwamini, Hema Magge

**Affiliations:** 1 Partners In Health/Inshuti Mu Buzima (PIH/IMB), P.O. Box 3432, Kigali, Rwanda; 2 Ministry of Health, Kigali, Rwanda; 3 Department of Global Health and Social Medicine, Harvard Medical School, Boston, USA; 4 Brigham and Women’s Hospital, Boston, USA; 5 Boston Children’s Hospital, Boston, USA; 6 Institute for Healthcare Improvement, Addis Ababa, Ethiopia

**Keywords:** Neonatal mortality, Neonate, Low birth weight, Prematurity, Quality of care, Mortality rate

## Abstract

**Background:**

Despite worldwide efforts to reduce neonatal mortality, 44% of under-five deaths occur in the first 28 days of life. The primary causes of neonatal death are preventable or treatable. This study describes the presentation, management and outcomes of hospitalized newborns admitted to the neonatal units of two rural district hospitals in Rwanda after the 2012 launch of a national neonatal protocol and standards.

**Methods:**

We retrospectively reviewed routinely collected data for all neonates (0 to 28 days) admitted to the neonatal units at Rwinkwavu and Kirehe District Hospitals from January 1, 2013 to December 31, 2014. Data on demographic and clinical characteristics, clinical management, and outcomes were analyzed using median and interquartile ranges for continuous data and frequencies and proportions for categorical data. Clinical management and outcome variables were stratified by birth weight and differences between low birth weight (LBW) and normal birth weight (NBW) neonates were assessed using Fisher’s exact or Wilcoxon rank-sum tests at the α = 0.05 significance level.

**Results:**

A total of 1723 neonates were hospitalized over the two-year study period; 88.7% were admitted within the first 48 h of life, 58.4% were male, 53.8% had normal birth weight and 36.4% were born premature. Prematurity (27.8%), neonatal infection (23.6%) and asphyxia (20.2%) were the top three primary diagnoses. Per national protocol, vital signs were assessed every 3 h within the first 48 h for 82.6% of neonates (*n* = 965/1168) and 93.4% (*n* = 312/334) of neonates with infection received antibiotics. The overall mortality rate was 13.3% (*n* = 185/1386) and preterm/LBW infants had similar mortality rate to NBW infants (14.7 and 12.2% respectively, *p* = 0.131). The average length of stay in the neonatal unit was 5 days.

**Conclusions:**

Our results suggest that it is possible to provide specialized neonatal care for both LBW and NBW high-risk neonates in resource-limited settings. Despite implementation challenges, with the introduction of the neonatal care package and defined clinical standards these most vulnerable patients showed survival rates comparable to or higher than neighboring countries.

## Background

Despite efforts to reduce neonatal mortality globally, in 2015, nearly half of the 5.9 million under-five deaths occurred in the first 28 days of life [1] and eight of the 10 countries with the highest neonatal mortality rates are in sub-Saharan Africa (SSA) [2]. The main clinical causes of death include prematurity, infection, inadequate management of complications of pregnancy and delivery, and lack of quality care immediately after birth [1–4]. The majority of these deaths are preventable through evidence-based clinical interventions, such as antibiotic administration [5], oxygen therapy, continuous positive airway pressure [6] and caffeine treatment [7, 8]. However, implementing these interventions in resource-limited settings can be challenging due to health system constraints, including limited equipment, lack of standardized protocols to guide neonatal management, insufficient training and support for clinical staff, and the shortage of pediatricians and neonatologists [9, 10].

In Rwanda, there has been a tremendous reduction in under-five mortality, which fell from 196 per 1000 live births in 2000 to 50 per 1000 live births in 2015, making the country one of the few in SSA to achieve the Millennium Development Goals for child mortality [11–14]. In addition, the neonatal mortality rate significantly declined from 41 per 1000 live births in 1990 to 17 per 1000 live births in 2016 [2]. Although national surveillance systems are able to provide population level data on neonatal survival [15], little data exists on the treatment and quality of care provided to high-risk newborns at health facilities, as well as predictors of clinical success in Rwanda or other countries in SSA. One study conducted in a rural Rwandan district hospital reported that over 60% of neonatal deaths occurred at presentation or shortly after admission and could be attributed in part to the lack of trained staff and lack of standard care practice supported by protocols [16]. Subsequently, the researchers identified the need for training staff and establishing protocols as vital for improving neonatal care.

The Rwanda Ministry of Health (MOH) developed a national neonatal care protocol and standards in partnership with a number of organizations, including Partners In Health/Inshuti Mu Buzima (PIH/IMB) - an international non-profit organization committed to improving health services in impoverished communities - and specialists from Boston Children’s Hospital in Boston, USA. The neonatal care package was nationally adopted in 2012 with the goal of providing quality care to sick and preterm/low birth weight infants in rural district hospitals which lack specialist physicians [17, 18]. The protocol was initially implemented and tested in the neonatal units of two rural PIH/IMB supported MOH district hospitals in 2010–2011. Details on the development and implementation of the newborn medicine program have been reported previously in Hansen et al’s 2015 study [18]. The protocol implementation included roll-out of standardized medical records, quality indicators, and corresponding training materials. Here, we describe the presentation, clinical management, and outcomes of neonates after approximately 2 years of implementation of the neonatal care package. Neonates admitted between January 2013 and December 2014 to the neonatal units of the two rural district hospitals where the care package was first introduced were included. We also compared outcomes between low birth weight (LBW) and normal birth weight (NBW) neonates to detect any differences that may exist in the delivery of care to these patients. We aimed to highlight successes and gaps in the implementation of the national neonatal care package that could support quality care provision for neonates in Rwanda and similar settings.

## Methods

### Study design and setting

This retrospective cross-sectional study included neonates admitted to the neonatal units of PIH/IMB supported MOH Rwinkwavu and Kirehe District Hospitals (RDH, KDH). Both hospitals are located in rural areas of the Eastern Province of Rwanda and serve a total population of approximately 550,000 [19]. The initial roll-out involved international neonatal physician and nurse specialist support. After introduction, routine technical support has included support for training and ongoing mentorship of general practitioners and nurses working in the neonatal units by PIH/IMB-employed Rwandan nurse mentors. Visiting specialist physicians supporting PIH/IMB’s medical education mission provided intermittent support. Additionally, PIH/IMB provided targeted support for essential equipment and consumables as part of health system strengthening.

At either of these two district hospitals, about 2700 to 2800 deliveries per year are recorded and roughly over 90% are referrals from health centers in the district hospitals’ catchment areas. The average facility delivery rate in the Eastern Province is 88.8% [15]. When neonates are born in clinical distress or exhibit risk factors such as LBW, prematurity, sepsis or birth asphyxia, they are transferred to a neonatal unit for ongoing clinical management.

### Neonatal unit structure and function

At the time of this study, seven to ten certified nurses or midwives staffed each neonatal unit. During the day shift, two nurses/midwives worked in the neonatology unit and one general practitioner (GP) supervised both the pediatric and neonatal units. Overnight, one to two GPs staffed the hospital and nurses/midwives in the neonatal units called them if in need of assistance. On average, there were 10 to 15 neonates in the neonatal unit at each hospital per day.

Training and on-site mentorship were provided intermittently throughout the study period by an expert physician and nurse trainers to the physicians and nurses staffing the units. Available equipment in the neonatal units included beds for kangaroo mother/skin-to-skin care, syringe pumps, incubators, radiant warmers and phototherapy units since 2011 and bubble continuous positive airway pressure (bCPAP) machines had recently been introduced in January 2013.

### Study population, sources of data and analysis

All neonates aged 0 to 28 days and admitted to the hospitals between January 1, 2013, and December 31, 2014 were included in our analysis. Data was extracted from patients’ medical files using a standardized neonatal data collection form and entered into an electronic database by hired and trained non-clinical data officers supporting hospital monitoring and evaluation. Before data collection the officers completed a two-day orientation and training that focused on specific clinical terms used in the neonatal units and how to read patients’ medical files and abstract data from the file to the data collection form. The data were extracted over a one-month period of time.

We analyzed a subset of data that contained neonatal demographic and clinical characteristics, clinical management and outcome information. The clinical management variables are based on the quality indicators, which were originally drafted for the protocol according to expert opinion and literature review and later modified to address topics identified as challenging by hospital clinical staff. The indicators aim to monitor key aspects of neonatal care provision per protocol, with a focus on the needs of sick, LBW and preterm neonates [17, 18]. The indicators tracked the monitoring of vital signs, thermoregulation, hypoglycemia, administration of antibiotics for infectious diseases, fluid electrolytes and nutrition, and respiratory distress and included targets for high quality (Table 1). Cut-off point for hypoglycemia changed during the study period. Originally, low blood sugar was defined as less than 40 mg/dl. The definition was eventually changed to be less than 45 mg/dl in order to support more cautious clinical management. It took time for the new definition to be applied, so for the purposes of our analysis we used the range of less than 40 or less than 45 mg/dl to define low blood sugar.

**Table 1:** Quality indicators definitions and targets

Category	Indicator Definitions	Targets
Vital signs	Percent of patient records in which vital signs are documented on average every 3 h within the first 48 h of admission	*15 (every 3 h × 48 h with possibility of one less on day of admission) per patient and 80% overall.*
Thermoregulation	Percent of neonates who have first temperature documented within 30 min of admission to neonatal unit	*80%*
	Percent of neonates with documented first temperature after admission < 36.0 °C having temperature improve to > 36.0 °C in ≤2 h	*80%*
Hypoglycemia^a^	Percent of neonates with documented blood sugar < 40- < 45 mg/dL who had blood sugar level improve to > 40- > 45 mg/dL within 1 h	*80%*
Infectious disease	Percent of neonates who received antibiotics (ampicillin and gentamicin) at correct dose and interval for first 24 h of therapy	*80%*
Fluid electrolytes and nutrition	Percent of neonates admitted to neonatal unit within first 48 h of life and remain in unit until at least 2 weeks of age who regain their birth weight by < 2 weeks of age	*80%*
Respiratory	Percent of neonates with BW < 1.5 kg or GA < 33 weeks for whom methylxanthine treatment (caffeine or aminophylline) is prescribed	*80%*
	Percent of preterm/LBW neonates eligible for CPAP who are started on CPAP within 2 h of life (eligibility criteria: BW < 2 kg or GA < 33 weeks and any degree of respiratory distress - O_2_ saturation ≤ 90% oxygen requirement and/or RR ≥50 and/or grunting/flaring/retractions)	*90%*

^*o*^*C* degrees centigrade, *mg/dL* milligrams per deciliter, *BW* birth weight, *LBW* low birth weight, *kg* kilogram, *GA* gestational age, *O2* Oxygen, *RR* respiratory rate, *CPAP* continuous positive airway pressure

^a^Cut-off point for hypoglycemia changed during the study period. Originally, low blood sugar was defined as less than 40 mg/dl. The definition was eventually changed to be less than 45 mg/dl. It took time for the new definition to be applied, so for the purposes of our analysis we used the range of less than 40 or less than 45 mg/dl to define low blood sugar

For this study, the antibiotic indicator is limited to antibiotics provided in the first 24 h of therapy. For routine monitoring and evaluation, it was most feasible to assess all charts with antibiotic provision, rather than algorithmic exclusions of those who were ruled out within 72 h. Additionally, medication safety is a critical issue, particularly when introducing treatments in a neonatal population which can require dilution calculations. Therefore, the routinely monitored indicator was adapted to the version included here. Correct dose and interval for the first 24 h of therapy was determined according to the national neonatal protocol [17]. The actual dose administered was compared to the calculated correct dose using the neonate’s birth weight, and administration interval was calculated using the time of medication administration as documented. In the two participating hospitals, the quality indicators were reviewed quarterly to monitor progress and inform quality improvement initiatives. All extracted data was verified for accuracy and completeness during routine audits performed by the MOH’s and PIH/IMB’s monitoring and evaluation teams.

We report demographic and clinical characteristics, clinical management and outcome variables using frequencies and proportions for categorical data and medians with interquartile ranges (IQR) for continuous data. We stratified the data by birth weight and used Fisher’s exact tests for categorical variables and Wilcoxon rank-sum tests for continuous variables to compare clinical management and outcomes variables between LBW and NBW neonates at the α = 0.05 significance level. NBW included neonates with a birth weight of ≥2500 g. LBW included neonates with a birth weight < 2500 g, with the sub-categories of extremely LBW neonates (< 1000 g), very LBW neonates (1000 to 1499 g) and LBW neonates (1500 to 2499 g). Although not used for stratification, gestational age is reported for some variables and was categorized as term (≥37 weeks) and preterm (<37 weeks). Outcomes included the number of neonates discharged, transferred, absconded (defined as leaving against medical advice) and deceased at the end of the study period, as well as weight at discharge and length of stay in the neonatal unit. Missing data were analyzed using a pairwise deletion for the missing data at random. Data were analyzed using Stata v13 (College Station, TX: StataCorp LP).

## Results

A total of 1723 neonates were admitted to the two district hospital neonatology units; 49.7% (*n* = 856) to Kirehe and 50.3% (*n* = 867) to Rwinkwavu (Table 2), respectively. Admission age was recorded for 1684 neonates; 88.7% (*n* = 1493) were admitted within the first 48 h of life and 58.4% (*n* = 949 of 1624) were males. Birth weight was recorded for 1518 neonates and gestational age recorded for 1528; 46.2% (*n* = 501) were LBW and 53.8% (*n* = 817) were NBW, and 36.4% (*n* = 556) were preterm and 63.6% (*n* = 972) were term. The top three primary diagnoses (among the 1663 neonates with recorded diagnoses) were prematurity (27.8%, *n* = 463), neonatal infection (23.6%, *n* = 392), and asphyxia (20.2%, *n* = 336).

**Table 2:** Socio-demographic and clinical characteristics of neonates admitted to neonatology units at two rural district hospitals in Rwanda (*N* = 1723)

	n	%
Hospital		
Kirehe	856	49.7
Rwinkwavu	867	50.3
Age at admission (days)	*N* = 1621	
< 1	1140	70.3
1–3	310	19.1
4–7	54	3.3
8–28	117	7.2
Admitted in first 48 h of life	*N* = 1684	
Yes	1493	88.7
No	191	11.3
Gender	*N* = 1624	
Male	949	58.4
Female	675	41.6
Birth weight (grams)	*N* = 1518	
Low birth weight (< 2500)	701	46.2
LBW (≥1500- < 2500)	528	34.8
Very LBW (≥1000- < 1500)	139	9.2
Extremely LBW (< 1000)	34	2.2
Normal birth weight (≥2500)	817	53.8
Gestational age (weeks)	*N* = 1528	
Preterm (<37)	556	36.4
Preterm (≥33 to < 37)	391	25.6
Very preterm (< 33)	165	10.8
Term (≥37)	972	63.6
Primary diagnosis	*N* = 1663	
Prematurity	463	27.8
Neonatal infection	392	23.6
Asphyxia at birth/low APGAR score/HIE	336	20.2
Respiratory distress/apnea	113	6.8
Low birth weight	95	5.7
Poor feeding	51	3.1
Malformation	36	2.2
Convulsion	28	1.7
Pneumonia	21	1.3
Jaundice	13	0.8
Hypothermia	10	0.6
Hypoglycemia	8	0.5
Others	97	5.8

*LBW* Low birth weight, *APGAR* A measurement of Appearance, Pulsation, Grimace, Activity and Respiration, *HIE* Hypoxic Ischemic Encephalopathy

For clinical management during a hospital stay, 82.6% (*n* = 965 of 1168) of neonates had their vital signs checked and documented at least 15 times within the first 48 h of their hospital admission (Table 3). For thermoregulation, 55.0% (*n* = 812 of 1476) of neonates had their initial temperature measured within 30 min of admission; 29.4% (*n* = 435 of 1480) had an initial temperature below 36 °C recorded, and 38.8% (*n* = 156 of 402) had an initial temperature below 36 °C improve to be greater than 36 °C within 2 h of the time the initial temperature was taken. Hypoglycemia was also assessed; 3.6% (31 of 871) of neonates had low blood sugar levels (either < 40 or < 45 mg/dl) and 16.7% (*n* = 2 of 12) neonates with low blood sugar improved to have a blood sugar measurement of either > 40 or > 45 mg/dl documented within the hour.

**Table 3:** Clinical management and interim outcomes of neonates with birth weight recorded upon admission to neonatology units at two rural district hospitals in Rwanda (*N* = 1518)

	All neonates (*N* = 1518)		Low birth weight (< 2500 g) (*N* = 701)		Normal birth weight (≥2500 g) (*N* = 817)		*p*-value
	n	%	n	%	n	%	
Vital signs							
Vital signs checked every 3 h for the first 48 h of admission	*N* = 1168		*N* = 556		*N* = 612		
Yes (≥15 times)	965	82.6	478	86.0	487	79.6	0.004
No (< 15 times)	203	17.4	78	14.0	125	20.4	
Thermoregulation							
Initial temperature measured within 30 min of admission	*N* = 1476		*N* = 683		*N* = 793		
Yes	812	55.0	367	53.7	445	56.1	0.373
No	664	45.0	316	46.3	348	43.9	
Initial temperature < 36 °C	*N* = 1480		*N* = 684		*N* = 796		
Yes	435	29.4	249	36.4	186	23.4	< 0.001
No	1045	70.6	435	63.6	610	76.6	
If initial temperature < 36 °C, temperature improved from < 36 °C to > 36 °C within 2 h	*N* = 402		*N* = 234		*N* = 168		
Yes	156	38.8	99	42.3	57	33.9	0.097
No	246	61.2	135	57.7	111	66.1	
Hypoglycemia							
Low blood sugar (< 40/< 45 mg/dl)	*N* = 871		*N* = 418		*N* = 453		
Yes	31	3.6	21	5.0	10	2.2	0.028
No	840	96.4	397	95.0	443	97.8	
If blood sugar low, improved to > 40/> 45 mg/dl within 1 h	*N* = 12		*N* = 7		*N* = 5		
Yes	2	16.7	1	14.3	1	20	> 0.999
No	10	83.3	6	85.7	4	80	
Infectious diseases							
Received antibiotics (ampicillin and gentamicin)^a^	*N* = 334		*N* = 69		*N* = 265		
Yes	312	93.4	68	98.6	244	92.1	0.053
No	22	6.6	1	1.4	21	7.9	
Ampicillin received at correct dose and interval	*N* = 301		N = 66		*N* = 235		
Yes	204	67.8	36	54.6	168	71.5	0.009
No	97	32.2	30	45.4	67	28.5	
Gentamicin received at correct does and interval	*N* = 286		*N* = 64		*N* = 222		
Yes	146	51.0	23	35.9	123	55.4	0.006
No	140	49.0	41	64.1	99	44.6	
Fluid electrolytes and nutrition							
Regained birth weight within 2 weeks^b^	*N* = 225		*N* = 169		*N* = 56		
Yes	126	56.0	88	52.1	38	67.9	0.044
No	99	44.0	81	47.9	18	32.1	
Respiratory distress							
Received caffeine^c^			*N* = 548				
Yes			206	37.6			
No			342	62.4			
Received oxygen therapy	*N* = 1491		*N* = 690		*N* = 801		
Yes	603	40.4	278	40.3	325	40.6	0.916
No	888	59.6	412	59.7	476	59.4	
Method of oxygen therapy	*N* = 540		*N* = 247		*N* = 293		
Mask/nasal cannula	449	83.1	185	74.9	264	90.1	< 0.001
bCPAP	91	16.9	62	25.1	29	9.9	
If eligible, received bCPAP^d^			*N* = 107				
Yes			13	12.2			
No			94	87.8			
Duration of oxygen therapy in hours	Median	IQR	Median	IQR	Median	IQR	
Mask/nasal cannula	24	20, 96	48	24, 120	24	14, 72	0.050
bCPAP	60	24, 120	72	24, 144	48	24, 72	0.093

^a^For neonates with primary diagnosis of infection; ^b^Restricted to neonates that were hospitalized for at least 2 weeks; ^c^Restricted to preterm neonates (< 33 weeks) or LBW (< 1500 g); ^d^Restricted to preterm (< 33 weeks) or LBW (< 1500 g) neonates with any sign of respiratory distress

^*o*^*C* degrees centigrade, *gm/dl* grams per decilitre, *bCPAP* bubble continuous positive airway pressure, *IQR* inter-quartile range

Of the 334 neonates with a primary diagnosis of infection, 93.4% (*n* = 312) received antibiotics. Among the 301 neonates who had data recorded on ampicillin administration and the 286 neonates who had data recorded on gentamicin administration, 67.8% (*n* = 204) and 51.0% (*n* = 146) received the correct dose at the correct time interval, respectively. Of 225 neonates who stayed in the hospital for at least 14 days, 56.0% (*n* = 126) regained their birth weight within the 2 weeks. With regard to respiratory support, 40.4% (*n* = 603) of 1491 neonates received oxygen therapy. Of 540 neonates with oxygen therapy method documented, 83.1% (*n* = 449) received mask or nasal cannula and 16.9% (*n* = 91) received bCPAP. Of the 107 preterm/low birth weight neonates meeting eligibility criteria for bCPAP (born < 1500 g or < 33 weeks and showing any signs of respiratory distress), 12.2% (*n* = 13) received bCPAP. Among the 548 neonates born < 1500 g or < 33 weeks (meeting eligibility criteria), 37.6% (*n* = 206) received caffeine citrate.

When results were stratified by birth weight, there was no evidence of differences in clinical management between LBW and NBW neonates for the following variables: initial temperature measured within 30 min of admission (*p* = 0.373), improvement in initial temperature from < 36 °C to > 36 °C (*p* = 0.097), improvement in low blood sugar levels to normal levels (*p* > 0.999), and administration of oxygen therapy (*p* = 0.916) (Table 3).

However, we observed significant differences in the clinical management of LBW and NBW neonates for a number of variables. Vital signs were checked at least 15 times within the first 48 h of admission for 86.0% (*n* = 478 of 556) of LBW neonates and 79.6% (*n* = 487 of 612) of NBW neonates (*p* = 0.004). Antibiotics were administered to 98.6% (*n* = 68 or 69) of LBW neonates with infection and 92.1% (*n* = 244 of 265) of NBW neonates with infection (*p* = 0.053). Correct dosage and interval of ampicillin was provided to 54.6% (*n* = 36 of 66) LBW neonates and 71.5% (*n* = 168 of 235) NBW neonates (*p* = 0.009), and to 35.9% (*n* = 23 of 64) LBW neonates and 55.4% (*n* = 123 of 222) NBW neonates for gentamicin (*p* = 0.006). Approximately half (52.1%, *n* = 88 of 169) of LBW neonates regained birth weight within 2 weeks, compared to 67.9% (*n* = 38 of 56) of NBW neonates (*p* = 0.044). For method of oxygen therapy, LBW neonates were more likely to receive bCPAP compared to NBW neonates (25.1%, *n* = 62 of 247 and 9.9%, *n* = 29 of 293, respectively, *p* < 0.001). LBW neonates received oxygen for a longer duration, whether on mask/nasal cannula (median = 48 h, IQR: 24–120, *p* = 0.050) or bCPAP (media*n* = 72 h, IQR: 24–144, *p* = 0.093) compared to NBW neonates, who used mask/nasal cannula for a median of 24 h (IQR 14–72) and bCPAP for a median of 48 h (IQR 24–72, *p* = 0.093).

Overall, 83.3% (*n* = 1162) of the neonates were discharged, 13.3% (*n* = 185) died, 2.3% (*n* = 32) transferred and 0.5% (n = 7) absconded (Table 4). The top three primary diagnoses among the 183 deceased neonates with primary diagnosis recorded were asphyxia (36.1%, *n* = 66), prematurity (29.5%, *n* = 54), and respiratory distress (13.1%, *n* = 24). There was no evidence of differences in outcomes between LBW and NBW neonates (*p* = 0.131). The overall median length of stay in the neonatal unit was 5 days (IQR: 2–10). The length of stay was significantly longer for LBW neonates (median = 7 days, IQR: 2–14) compared to NBW neonates (median = 4 days, IQR: 2–7, *p* < 0.001). This remained true when stratified by those who survived to discharge (median = 8 days, IQR: 3–18 vs. median = 5, IQR: 2–8, *p* < 0.001) and those who did not (median = 2 days, IQR: 0–2 vs median = 1 day, IQR: 0–2, *p* = 0.003). Of the neonates who were discharged, 477 were low birthweight at admission, and 94.1% (*n* = 449) remained under 2500 g when they were discharged, while of the 536 neonates of normal birthweight at admission, 12.3% (*n* = 66) were under 2500 g at discharge (*p* < 0.001).

**Table 4:** Outcomes of neonates with birth weight recorded upon admission to neonatology units at two rural district hospitals in Rwanda (*N* = 1518)

	All neonates (*N* = 1518)		Low birth weight (< 2500 g) (*N* = 701)		Normal birth weight (≥2500 g) (*N* = 817)		*p*-value
	n	%	n	%	n	%	
Outcome	*N* = 1386		*N* = 626		*N* = 760		
Discharged	1162	83.3	522	83.4	640	84.2	0.131
Transferred	32	2.3	11	1.8	21	2.8	
Absconded	7	0.5	1	0.2	6	0.8	
Died	185	13.3	92	14.7	93	12.2	
Length of stay^a^	Median	IQR	Median	IQR	Median	IQR	
All neonates	5	2, 10	7	2, 14	4	2, 7	< 0.001
Discharged	6	2, 11	8	3, 18	5	2, 8	< 0.001
Transferred	3	0.5, 9.5	3.5	0, 11	2.5	0.5, 7.5	0.722
Absconded	7	1, 15	21	21, 21	5	1, 8	0.134
Died	1	0, 3	2	1, 4	1	0, 2	0.003
Weight at discharge (grams)^b^	*N* = 1013		*N* = 477		*N* = 536		
< 2500	515	50.8	449	94.1	66	12.3	< 0.001
≥ 2500	498	49.2	28	5.9	470	87.7	

^a^Only reported for neonates that had an admission date and outcome date or age at admission and age at discharge; ^b^Limited to neonates who were discharged

## Discussion

Neonatal care provision in rural resource-limited settings is a challenge for many countries in SSA and in the early stages of introduction [18–25]. Hansen et al. (2015) showed that while neonatal care may be considered a specialized clinical service, it can be standardized and implemented in rural district hospitals in Rwanda [18]. Here, we show clinical management and quality of neonatal care in two rural district hospitals that were guided by this neonatal care package. Our study had a number of key results.

First, we found that the demographic and clinical characteristics of our neonatal sample were similar to those reported in other studies. Consistent with studies showing higher and earlier hospital admission rates for neonates within the first 7 days of birth and higher morbidity rates among male neonates [2, 20, 26, 27], most neonates in our study were admitted to the neonatal units within the first 48 h after birth and were males. Prematurity, infection and asphyxia were the top three causes of infant illnesses, mirroring findings reported in other parts of SSA [1, 20, 27, 28]. These patterns show that more research into the underlying causes of neonatal illness in resource-limited settings is needed, as are interventions that can address these persistent challenges.

Second, our assessment of clinical management and outcome variables shows that it is possible to provide care to high-risk neonates in this setting with reasonable patient outcomes, particularly when compared to those of other countries in the region [27]. A study on the use of medicines in 104 developing and transitional countries from 1990 to 2009 found that the percentage of patients receiving antibiotics increased from 45 to 54% over this 20-year period [29] and the management of prematurity-related complications using antibiotics was only 50% [27]. Additionally, the majority of LBW neonates, who are at the highest risk of morbidity, were more likely to receive care according to protocol compared to NBW neonates in a number of clinical domains, including close monitoring (vital sign measurement) and duration on oxygen therapy, which are important interventions to reduce clinical deterioration in this population [5, 30]. LBW neonates also stand to gain the greatest impact from bCPAP for the treatment of respiratory distress syndrome [5, 21] and their higher rates of bCPAP use and longer duration on oxygen therapy showed possible prioritization of their needs by health care workers, particularly in a staff-constrained environment. The transfer rate for neonates was low and impressively similar to a study conducted at a Rwandan provincial hospital (which is supposed to have a higher level of care than district hospitals) which reported a drop-in neonate transfer rates from 50 to 2% in 1 year after a series of quality improvement interventions were implemented, including introduction of standardized treatment procedures [16].

Finally, and most importantly, the overall neonatal unit survival rate for both LBW and NBW neonates was higher than results reported from similar settings and did not differ across birth weight categories [23, 26, 31]. This study is descriptive and limits any assessment of causality; however, given that no standardized care for sick and preterm infants was provided before the establishment of the neonatal care unit and these infants were previously being referred to the one national referral hospital in the country, it demonstrates that neonatal care can be provided in a decentralized manner with the appropriate investment in neonatal unit establishment, vastly improving population access to this essential service. Availability and ease of protocol use, presence of essential equipment and medications, along with intermittent training and mentorship for providers likely influenced the quality of care for high-risk neonates in these hospitals [32, 33]. Asphyxia was a leading diagnosis among those who died, which could indicate a greater need to target improvement efforts during delivery and immediately after birth and underscore the need for interdepartmental coordination within the hospital across maternity, delivery, and neonatal units. Further research assessing the impact of the neonatal care package on population level neonatal mortality, as well as assessment of the optimal content and level of complexity for rural district hospitals is warranted.

Despite these successes, there is still a need to improve treatment practices to meet quality indicator targets and further improve neonatal outcomes. These targets were intentionally set high to serve as a goal for quality improvement efforts. Based on our results, management of vital signs measurement, weight gain, administration of antibiotics and thermoregulation measurement of temperature within the first 30 min of admission were close to the targets, while other indicators, such as improved temperature within 2 h of admission, caffeine administration, improved blood sugar levels and weight gain were not. These results can be used to inform the design and goals of quality improvement initiatives so they can be most effective. For example, while LBW infants had relatively strong outcomes, a low percentage of the bCPAP-eligible LBW/preterm neonates received bCPAP therapy, indicating further potential gains in neonatal outcomes that could be made. While this gap could be exaggerated due to the fact that bCPAP was introduced in first month of the study period and may have improved with increasing health care worker comfort and practice over time during the study period, a gap in bCPAP implementation was also noted by a second bCPAP-focused study from an overlapping time period [34]. Therefore, this area has been targeted for quality improvement since the completion of this study.

In addition, while antibiotics were administered to neonates diagnosed with infections, there is room for improvement in the timing and dosage of these antibiotics. As these data were used for quarterly data review with the clinical teams, quality improvement problem analyses found a number of challenges with hospital level neonatal antibiotic administration. The dose has to be selected based on gestational age/birth weight and a weight-based dose had to be correctly calculated. Some providers new to neonatal care, or not providing neonatal care routinely, found this selection challenging, and more NBW neonates received correct administration of gentamicin. Staff shortages and high patient volume could be challenges to administering antibiotics on-time. There is also room for improvement around documentation and recordkeeping. Further exploration into the issue should be pursued by asking the neonatal nurses about the documentation processes they follow and challenges they face. Observation of documentation practices can also be done if possible. Strategies for improving documentation should be designed and implemented as a collaborative effort between nurses and researchers to better support uptake and sustainability.

Our results also show that quality improvement efforts need to be directed at NBW neonates as well. We found that 12% of neonates admitted to the hospital with a normal birthweight were discharged weighing less than 2500 g. While this could be focused among neonates born just above the LBW cut-off experiencing normal weight loss in the first few days of life with short-course hospitalizations, this may indicate that supporting neonatal growth and nutrition was challenging. Taking the full context into consideration, this may be due to a family’s inability to pay for the costs of hospitalization or because parents are unable to leave other children at home or be absent from work to be in the hospital. Therefore, this finding warrants further investigation into how to support families comprehensively, and more interventions, such as social support packages that can address financial challenges, are needed to ensure these high-risk vulnerable neonates get the treatment they need to thrive.

Health systems challenges may have also influenced the ability to reach certain indicator targets. Not being able to apply to full protocol due to staffing shortages, high turnover of trained staff, misaligned rotations where providers not trained in neonatal care are assigned to the neonatal unit and stock outs of drug and laboratory reagents may have impacted the quality of care provided in the neonatal units [4, 28]. We recommend implementation of quality improvement projects that target the national indicators, accurate and timely documentation of management and outcomes, revision of the rotation system to ensure the nurses staffing the neonatal unit have necessary training and skills for neonatal management and harmonizing internal transfers of neonates to ensure continuity of care as activities that can address the challenges we see in our neonatal units [35].

It is important to note that the care protocol was not introduced as an isolated document – when first introduced, it was paired with medical record introduction, quality indicators, and institutionalized training and mentorship. More broadly, will-building across hospital leadership and health care providers to establish a neonatal unit with designated physician and nursing staff and management was critical to providing a setting in which this introduction could have any measure of success. The district hospitals have both adopted district-based quality improvement initiatives in response to these data which were reviewed quarterly, including projects aimed at accurate and timely documentation of management and outcomes, revision of the rotation system to ensure the nurses staffing the neonatal unit have necessary training and skills for neonatal management, and coordination of internal transfer processes of neonates to ensure continuity of care [36].

This study has several limitations. First, as a retrospective cross-sectional study over a two-year time period, we were unable to assess variations in quality across time and potential relationships with intervening variables such as those related to constrained health systems and timing of introduction of new initiatives such as bCPAP listed previously. We do not have data for year-to-year variations in the indicators. Data variation over a longer period would have to take issues like staffing and policy changes into consideration, which were outside the scope of this study. Along the same vein, we chose not to examine trends in performance on the quality indicators because we chose to focus on the current level of performance. Trends in performance in relation to specific quality improvement interventions could be the subject of future studies. Additionally, some of the diagnoses, such as infection and asphyxia, require laboratory evaluations for definitive diagnosis that were not available at this district hospital level [22]. However, we believe the clinical management to be appropriate if the provider closely followed the guidance for clinical management of a given suspected diagnosis, as they are designed to leverage the diagnostic resources available in these low-resource settings. Finally, another limitation was missing data, which we believe was due to the high documentation burden placed on the nurses and physicians staffing the neonatal units. Approximately 12% of the records were missing data and not included in the analysis of clinical management and interim outcomes shown in Table 3. Results were reported only for neonates who had a birth weight recorded upon admission to the neonatology unit. While missing data limited the ability to which we were able to generalize our study findings, we are still able to present outcomes and discuss our experiences of treating small and sick neonates in accordance with a national neonatal care package. Periodic data quality checks and training with an emphasis on the importance of proper documentation in clinical charts could improve data quality. In addition, future plans to implement electronic medical record systems in the hospital units could also help address these data quality gaps.

## Conclusions

This study demonstrates the feasibility of specialized neonatal care in resource-limited settings supported by a standardized national package of services including infrastructure, training and supplies, as well as the implementation of a national neonatal protocol. LBW neonates received higher quality care compared to NBW and overall mortality rates were lower or comparable to other urban and tertiary hospital settings among both categories. However, gaps in care management remain that should be addressed in order to achieve further gains in morbidity and mortality. We recommend quality improvement efforts to address lagging indicators as well as continuous training and mentorship to ensure new providers are empowered with the tools necessary to provide high quality care to these vulnerable newborns and families.

## Abbreviations

bCPAP: Bubble continuous positive airway pressure
GP: General practitioner
IQR: Interquartile range
KDH: Kirehe District Hospital
LBW: Low birth weight
NBW: Normal birth weight
^o^C: Degrees centigrade
PIH/IMB: Partners In Health/ Inshuti Mu Buzima
RDH: Rwinkwavu District Hospital
MOH: Ministry of Health
SSA: Sub-Saharan Africa
